# Spheroidal carbonaceous particles are a defining stratigraphic marker for the Anthropocene

**DOI:** 10.1038/srep10264

**Published:** 2015-05-28

**Authors:** Graeme T. Swindles, Elizabeth Watson, T. Edward Turner, Jennifer M. Galloway, Thomas Hadlari, Jane Wheeler, Karen L. Bacon

**Affiliations:** 1 School of Geography, University of Leeds, Leeds, LS2 9JT; 2 Geological Survey of Canada, Calgary, Alberta, T2L 2A7.

## Abstract

There has been recent debate over stratigraphic markers used to demarcate the Anthropocene from the Holocene Epoch. However, many of the proposed markers are found only in limited areas of the world or do not reflect human impacts on the environment. Here we show that spheroidal carbonaceous particles (SCPs), a distinct form of black carbon produced from burning fossil fuels in energy production and heavy industry, provide unambiguous stratigraphic markers of the human activities that have rapidly changed planet Earth over the last century. SCPs are found in terrestrial and marine sediments or ice cores in every continent, including remote areas such as the high Arctic and Antarctica. The rapid increase in SCPs mostly occurs in the mid-twentieth century and is contemporaneous with the ‘Great Acceleration’. It therefore reflects the intensification of fossil fuel usage and can be traced across the globe. We integrate global records of SCPs and propose that the global rapid increase in SCPs in sedimentary records can be used to inform a Global Standard Stratigraphic Age for the Anthropocene. A high-resolution SCP sequence from a lake or peatland may provide the much-needed ‘Golden Spike’ (Global Boundary Stratotype Section and Point).

The Anthropocene has become a term widely adopted by both the scientific community and the media^1^,^2^. It reflects the current time of the Earth’s history when human activities have become one of the dominant forces shaping the planet implying that a new geological time division may be required. There has been much debate over the timing of the Anthropocene; some authors have used archaeological evidence to suggest that the rise of human impacts began in the early to mid-Holocene^3^, ~2 millennia ago^4^, or from the time of the industrial revolution^1^. However, mounting evidence suggests that human impacts on the planet at these times were diachronous and highly spatially variable^5^. There is rising support for the base of the Anthropocene to be placed at ca. AD 1950 that approximates the ‘Great Acceleration’, a time of rapidly increasing and globally-widespread anthropogenic impacts on planet Earth. This includes unprecedented burning of fossil fuels leading to a rapid rise in global atmospheric CO_2_, deployment of nuclear weapons, and pollution from industrial, agricultural and domestic processes^5^,^6^. There is a need for a stratigraphic marker that reflects the significant global impact of humans on Earth and defines the Anthropocene. This stratigraphic marker must represent the onset of the Anthropocene in marine and terrestrial sediments and ice, be present across the globe, and be related to the types of human impacts that characterize the Anthropocene. Anthropogenic soils^4^, chemical tracers^7^, and radionuclides^5^ have been proposed as Anthropocene markers. However, many of these are diachronous, regionally variable, occur at the wrong time, or require a complex analytical procedure to decipher. The Tambora 1815 volcanic event has also been proposed as a possible marker for dating the onset of the Anthropocene^8^. This volcanic event is registered in chemical profiles from Greenland and Antartica ice core records; however, tephra from this eruption is only found in Asia^9^. More importantly, it does not derive from human impacts on the environment that defines the Anthropocene.

An unambiguous ‘index fossil’ of the human activities that have changed the face of planet Earth in recent centuries is therefore needed. Spheroidal carbonaceous particles (SCPs) are a distinct component of black carbon only produced from the high-temperature (>1000 °C) combustion of fossil fuels (coal and oil) (Supplementary file). SCPs are produced as a by-product of energy production as well as heavy industry and have no natural sources in the Quaternary^10^. SCPs are highly abundant in areas close to pollution sources^11^,^13^, and are also found across the continents of planet Earth (Table 1). Importantly, they have also been recorded in remote areas distal from industrial sources including Greenland^14^, Svalbard^15^, Arctic Canada^16^,^17^ and Antarctica^18^. Several studies have shown that SCPs are correlated with other types of industrial pollution including sulphur and polycyclic aromatic hydrocarbons (PAHs)^13^,^19^. In addition, SCPs are also well-preserved in lake and marine sediments, peats and glacial ice as they are chemically inert, owing to their composition of elemental carbon^20^,^21^.

SCPs are suitable indicators for the Anthropocene for the following reasons:

1. They are present across the globe (Table 1);
2. They are an unambiguous marker of anthropogenic fossil fuel combustion that has changed the composition of our atmosphere and driven recent climate change^22^;
3. They record unprecedented impacts of human activity on the environment;
4. They are documented in ice cores, marine and terrestrial sediments;
5. They are easily extracted and identified by researchers.

Variation in the timing and extent of coal and oil usage are reflected in temporal differences of the first occurrence (First Occurrence Datum) of SCPs in different regions (Table 1). The peak (acme) in SCP concentration is also variable spatially, reflecting proximity to pollution sources. However, the rapid increase in SCPs reflects the rise to dominance of oil as the major fossil fuel source on Earth^22^ and mostly occurs in the mid-twentieth century across the globe (Fig. 1) – contemporaneous with the ‘Great Acceleration’^3^ and rapid increase in global population. The rapid increase in SCPs is thus a key chronostratigraphic marker for the Anthropocene because it is a global signature. We know of only two pre-Holocene occurrences of SCPs in the sedimentary record due to non-anthropogenic phenomena and they both correlate to significant geological timescale boundaries and mass extinctions. SCPs were derived from (I) the combustion of coal by flood basalts at the latest Permian extinction^23^; and (II) the combustion of fossil organic matter from bolide impact at the Cretaceous-Palaeogene boundary^24^. Furthermore, it has been suggested that the Cretaceous–Palaeogene examples are easily distinguished from modern SCPs due to a lower burn temperature resulting in lighter colouration of the particles^25^. We suggest that the appearance of SCPs in Anthropocene sediments will appear geologically instantaneous in the far future.

We propose that lake and/or peatland sequences with detailed SCP records should be used to inform either a Global Standard Stratigraphic Age (GSSA), or used as the Global Boundary Stratotype Section and Point (GSSP) for the Anthropocene. Many lakes and peatlands have continuous sedimentation/accumulation over this time period and deposited an adequate thickness of sediment to capture SCPs. In Britain for example, SCP records commonly begin between AD 1830–1860^25^, somewhat later than the earliest industrialisation. However, the rapid increase in SCPs (AD 1950–1960s) reflects the increased intensity of fossil fuel use in industry and power generation after the second world war that: (1) left an unambiguous expression in sedimentary records; and (II) reflects impact of global significance. The rapid increase in SCPs provides a marker of the point in time when human activities became globally unprecedented, rather than reflecting first intense industrialisation. An exceptional lake or peatland SCP record would need to be calibrated to a specific year using a marker horizon, such as an ash bed as close as possible to the rapid increase in SCPs. The deposition of the Hekla 1947 ash in Irish peatlands immediately prior to the rapid increase in SCPs may provide such a calibration^26^.

There are numerous secondary markers that could be used to mark the onset of the Anthropocene, including chemical signatures of anthropogenic pollution (e.g. PAHs, Pb, Hg), land degradation (e.g. dust from soil erosion), or changes to biodiversity such as extinctions of native biota or introductions of non-native species. However, these signals are not consistent globally. Biostratigraphic evidence of recent climate or human impacts in lakes and peatlands (e.g. microfossils such as chironomids, diatoms, testate amoebae, and non-native pollen) may also be used in some localities. Recent peats and sediments have been dated using radio-isotopes such as ^210^Pb, ^137^Cs and high-resolution ^14^C techniques, permitting precise dating of the rapid increase in SCPs.

To support our argument we provide a reference example from Malham Tarn Moss, a raised bog in the Yorkshire Dales, N. England (Fig. 2). A high-resolution SCP sequence combined with records of lead pollution and soil erosion (Fe, loss-on-ignition) are illustrated. These reflect increased land-use intensity, direct human impacts on the peatland and peatland response to climate change (water table depth reconstruction based on testate amoebae microfossils). This record illustrates the unprecedented human impacts on the environment in N. England after c. AD1950 which occurs alongside the rapid increase in SCPs. Our example clearly demonstrates the utility of SCPs as a defining stratigraphic marker for the Anthropocene.

## Methods

We carried out a detailed analysis of published literature to assess the occurrence of spheroidal carbonaceous particles (SCPs) in sediment and peat profiles and ice cores across the world (Table 1). All sources were compiled by country and continent and the established dates of the first occurrence and the onset of rapid increase of SCPs were noted (based on independent dating methods including ^210^Pb determinations and tephra). A Kernal density function was used to estimate the probability density function of the date of rapid increase in SCPs. Two adjacent cores from Malham Tarn Moss Yorkshire Dales, Northern England (54.0975946°, −2.1730828°) were taken using a Russian-type D section corer. One core was analysed for 21 chemical elements using a Cox Analytical Systems ITRAX X-ray fluorescence core scanner at 500 μm intervals to semi-quantitatively determine Pb and Fe content. The other core was divided into 1-cm contiguous sections for SCP analysis. SCP concentrations were analysed under high-powered microscopy following acid digestion and presented as *n* per g dry peat^27^. Calendar ages for the first occurrence, rapid increase, and peak concentration of SCP were assigned to the record^20^,^25^. Loss-on-ignition was determined using standard methods^28^. Water table depth reconstruction was carried out on subfossil testate amoebae using a transfer function based on a local training set^29^.

## Additional Information

**How to cite this article**: Swindles, G. T. *et al*. Spheroidal carbonaceous particles are a defining stratigraphic marker for the Anthropocene. *Sci. Rep.* **5**, 10264; doi: 10.1038/srep10264 (2015).

## Supplementary Material

Supporting Information Supplementary Figures 1-6

## Figures and Tables

**Figure 1:**
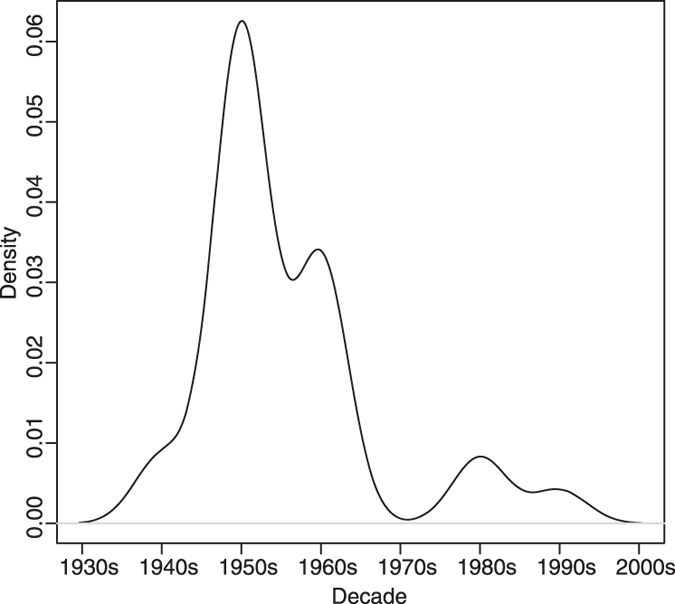
Kernel density plot of the decade of rapid increase in global SCPs (data are from Table 1). The plot illustrates that the rapid increase mainly occurs in the mid-twentieth century across the globe. The earliest decade was used in the case of the event spanning two decades (e.g. 1950s–1960s).

**Figure 2:**
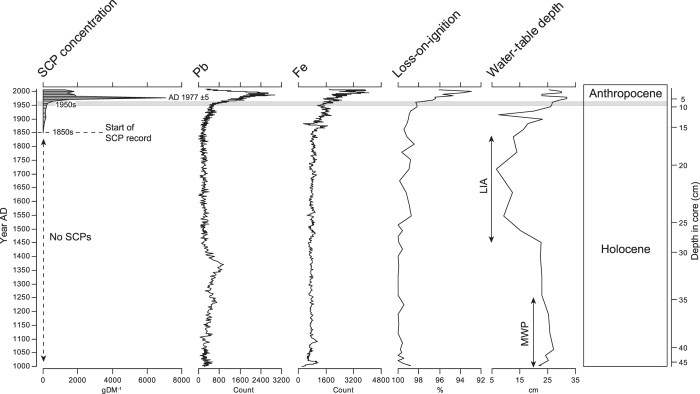
Spheroidal carbonaceous particle record from Malham Tarn Moss, a peat bog in the Yorkshire Dales, Northern England. The first occurrence of SCPs in the mid-19^th^ century reflects the onset of industrial combustion of coal at high temperature. The rapid increase in the 1950s reflects the increase in total energy production after the Second World War. Human impacts on Malham Tarn Moss become unprecedented at this time, including atmospheric deposition of Pb and soil erosion from intensive agricultural practices (reflected in the loss-on-ignition and Fe data from the peat bog) and a rapid increase in SCP deposition. The top of core represents the year of sampling (2009). The Medieval Warm Period (MWP) and Little Ice Age (LIA), marked by drier and wetter bog surface wetness respectively, are shown and the proposed Holocene–Anthropocene boundary of AD 1950 is illustrated by the grey line.

**Table 1:**
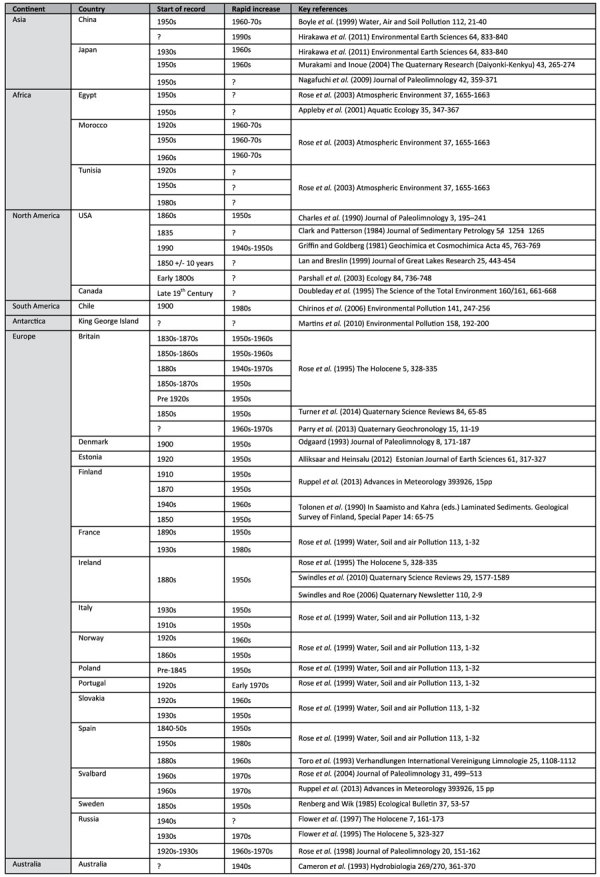
The global occurrence of spheroidal carbonaceous particles. The reported ages of the first occurrence and rapid increase of SCP concentration are provided with key references.
