# Supplementary material for: Spheroidal carbonaceous particles are a defining stratigraphic marker for the Anthropocene
Source: Sci Rep. 2015 May 28;5:10264. doi: 10.1038/srep10264 (PMC4603698; doi:10.1038/srep10264)
Supplement: Supporting Information — Supplementary Figures 1-6 [file srep10264-s1.doc]

**Spheroidal carbonaceous particles are a defining stratigraphic marker for the Anthropocene**

**Supplementary material**

Graeme T. Swindles^1*^, Elizabeth Watson^1^, T. Edward Turner^1^, Jennifer M. Galloway^2^, Thomas Hadlari^2^, Jane Wheeler^1^ and Karen L. Bacon^1^

^1^School of Geography, University of Leeds, Leeds, LS2 9JT

^2^Geological Survey of Canada, Calgary, Alberta, T2L 2A7

*Corresponding author

[g.t.swindles@leeds.ac.uk](mailto:g.t.swindles@leeds.ac.uk), +44 (0)11334 39127

**Characteristics of SCPs**

| **Category** | **Characteristic** |
| --- | --- |
| Shape | Spheroidal, but not spherical |
| Surface | Often lacy or pitted structures |
| Size | Mostly 2–20 𝜇m (diameter), largest particles can reach >50 𝜇m.  In very remote areas most particles are smaller than 10 𝜇m. |
| Colour | Black or dark brown |
| Other properties | Highly refractory, chemically inert, hydrophobic and thus fossilize well |

**▲Supplementary Table.** Characteristic features of spheroidal carbonaceous particles.

Photos of SCPs are available in the following paper:

<http://pixelrauschen.de/wbmp/media/map07/map_07_03.pdf>
